# Exploration of Milk and Dairy Products: Sensory, Physicochemical Characteristics and Processing Technologies

**DOI:** 10.3390/foods15183293

**Published:** 2026-09-17

**Authors:** Giuseppe Natrella, Michele Faccia

**Affiliations:** Department of Soil, Plant and Food Sciences, University of Bari, Via Amendola 165/A, 70126 Bari, Italy; michele.faccia@uniba.it

## 1. Introduction

Milk and dairy products have accompanied human diets for millennia, and their nutritional relevance—as sources of high-quality protein, essential fatty acids, vitamins and minerals—has never really been in question. However, consumers have changed their habits and perspective on foods. The modern vision of food is that is not only expected to simply nourish, but to do something for the person eating it (i.e., improve generally people health, such as lowering the risk of noncommunicable disease) [1]. Some examples of objective pursuit are the transition to a more “natural” ingredient list, finding strategies to formulate new foods including reintroduction of bioactive compounds which are typically lost during processing processes, and reducing the presence of some food component responsible of diseases; all of these aim to improve the quality and safety of foods. Thus, the following are needed to achieve this: (i) a faster way to detect cold-tolerant spoilage organisms before they cause losses; (ii) better tools for quantifying some sensory perceptions that are considered slippery, such as mouthfeel; (iii) formulation strategies that can improve nutritionally products, i.e., remove fat without modifying the sensory qualities that make a cheese or a yogurt worth eating. The research lines in this Special Issue cover many relevant topics, some of which include the aims mentioned above. From a farm perspective, supplementing dairy herds with essential oils [2,3] has already demonstrated an effect in reshaping milk fat composition and improving oxidative stability further down the chain. Concerning the production process, fortifying with prebiotics, probiotics or fiber [4] is advantageous for products that would have been difficult to justify commercially in the past (i.e., symbiotic camel milk powders, reduced-fat cheeses meeting nutrient-content claims). On the analytical side, multiplex qPCR [5] is starting to replace slower culture-based screening, which is usually time consuming, for quality control, while tribology and rheology [6,7,8] are giving researchers an instrumental handle on textures that used to be described only in terms of trained sensory panels.

This Special Issue, “Exploration of Milk and Dairy Products: Sensory, Physicochemical Characteristics and Processing Technologies,” was designed to engage with these types of problems. The six published papers propose many solutions, from herd management to processing to sensory measurement. Despite the studies relying on different experimental approaches and focusing on distinct dairy systems, they deepen our understanding of the factors that affect the quality and value of dairy products. The main findings of the contributions are summarized below.

## 2. Overview of the Published Articles

Early detection of microorganisms associated with dairy spoilage is essential for controlling quality throughout the milk supply chain. In Contribution 1, Yalew et al. developed and validated a real-time multiplex qPCR assay for the simultaneous detection and quantification of enzyme-producing psychrotrophic bacteria in raw milk. These microorganisms are particularly relevant because they can grow under refrigerated conditions and produce extracellular lipases and proteases that may remain active even after pasteurization. Their activity can subsequently lead to casein degradation, bitterness, and gelation problems in long-life dairy products. To address the issue, the authors designed specific primers and hydrolysis probes targeting conserved regions of the lipA gene, encoding triacylglycerol lipase, and the aprX gene, encoding an alkaline metalloprotease. The approach builds on established qPCR methodologies for dairy characterization [5] while providing a faster alternative to conventional culture-based procedures [8]. The assay showed high analytical sensitivity, with a limit of detection of 10^3^ CFU/mL in artificially contaminated UHT milk and 0.0002 ng/µL of genomic DNA. Its applicability was further demonstrated using naturally contaminated raw milk collected from commercial dairies. Overall, the method provides dairy processors with a rapid molecular tool for assessing the presence of potentially spoilage-associated bacteria, with results available considerably earlier than those obtained through conventional microbiological enumeration.

The Contribution 2, Herrera et al. focused on the preservation and valorization of local livestock breeds, which can contribute not only to biodiversity conservation but also to the sustainability and economic viability of traditional dairy production. Herrera et al. investigated artisanal hard goat cheeses produced from raw milk obtained from four Spanish autochthonous breeds: Cabra del Guadarrama, Malagueña, Florida, and Murciano-Granadina. The cheeses differed significantly in several physicochemical and microbiological characteristics, including fat and protein contents, pH, and moisture. Some of the most relevant differences were related to the nutritional characteristics of the lipid fraction. In particular, cheeses from the endangered Cabra del Guadarrama and Malagueña breeds showed a favorable lipid profile, including an omega-6/omega-3 ratio below 4 and relatively low atherogenic and thrombogenic indices. These characteristics are of interest in the context of nutritional quality and cardiovascular health. An additional strength of the study was the assessment of consumer acceptance under both blind and informed tasting conditions. Providing information about the use of milk from native breeds increased overall liking, particularly for the Guadarrama and Florida cheeses. The results therefore suggest that information about breed origin can influence consumers’ perception of traditional dairy products. More broadly, communicating the link between these products and local genetic resources could increase their market value while providing an additional incentive for maintaining endangered breeds and supporting the rural communities associated with their production.

The growing interest in functional foods has encouraged the development of dairy products designed to provide benefits beyond basic nutrition. Contribution 3, by Sakr and Barakat, explored this through the development of a freeze-dried probiotic fermented camel milk enriched with organic Ajwa date pulp (ADP). Camel milk was selected as the dairy matrix because of its distinctive nutritional composition and its potential suitability for consumers who have difficulty tolerating bovine milk. The researchers combined a commercial ABT-5 starter culture, containing *Lactobacillus acidophilus*, *Bifidobacterium bifidum*, and *Streptococcus thermophilus*, with or without *Lacticaseibacillus rhamnosus* B-1937. The fermented products were further supplemented with either 12% or 15% ADP, resulting in a formulation designed to combine probiotic microorganisms with the prebiotic and bioactive components supplied by the date pulp. The strategy is consistent with the increasing interest in combining dietary fibers and bioactive compounds in foods intended to support metabolic health. After freeze-drying, the formulations showed low water activity (0.196–0.226), together with improved dispersibility and reconstitution characteristics, which were associated with the composition of the date pulp. The supplemented products also maintained good probiotic viability during exposure to simulated gastric and intestinal conditions. In vitro assays further indicated dose-dependent inhibition of α-amylase and α-glucosidase, suggesting potential antidiabetic activity, while selective cytotoxic effects were observed against Caco-2 colon cancer cells. On the whole, these outcomes indicate that the combination of camel milk fermentation and Ajwa date pulp could be considered an interesting approach for developing stable, probiotic-rich functional dairy powders with potential nutritional and health-related applications.

In Contribution 4, Antonino et al. addressed the fat reduction in cheese while maintaining the characteristic texture and sensory properties of the full-fat product, a relevant technological challenge. The aim was achieved through a combined nutritional and technological approach. Whole-milk control Mozzarella (MC) was compared with a reduced-fat experimental cheese (MI), produced from milk obtained from cows orally supplemented with laurel essential oil (LEO) and fortified with 10% (*w*/*v*) long-chain inulin during the stretching phase. The dietary supplementation was intended to modify the fatty acid composition of milk, while the addition of inulin was used to compensate for some of the technological and sensory effects associated with fat reduction. Dietary strategies based on natural compounds such as essential oils have previously been investigated as a means of modifying milk fat composition and related sensory properties [2]. The incorporation of inulin resulted in a slightly longer manual stretching time, but the final cheese retained 3.31% fiber, allowing it to meet the European Union requirements for the “Source of Fiber” claim under Regulation (EC) No 1924/2006. Texture analysis showed that the inulin developed a hydrothermal micro-gel network within the cheese matrix, contributing to its structural properties and partially replacing some of the functions normally provided by milk fat. As a result, the MI cheese showed higher hardness and springiness during the 10-day storage period. The use of inulin as a texturizing agent and fat replacer is consistent with its previously reported applications in cheese technology [4]. At the same time, LEO supplementation modified the fatty acid composition of the milk and cheese, resulting in an approximately 40% increase in monounsaturated fatty acids (MUFAs) together with an increase in polyunsaturated fatty acids (PUFAs). These compositional changes were accompanied by differences in the sensory profile. Temporal Check-All-That-Apply (TCATA) analysis indicated that the inulin-fortified cheese maintained a relatively stable texture and structural profile throughout storage. The study therefore illustrates how nutritional intervention at the farm level can be combined with processing strategies to address both the nutritional and technological limitations of reduced-fat cheese.

Dietary supplementation of dairy animals with plant-derived compounds may also influence the stability and microbiological characteristics of the resulting dairy products. In Contribution 5, da Silva et al. investigated the effects of an essential oil blend (EOB), consisting of eucalyptus oil, peppermint oil, and menthol crystals, administered to lactating Jersey cows. The study was part of a broader interest in the use of essential oils in animal nutrition to modify milk composition and improve the oxidative stability of dairy products [3]. The evaluation of the physicochemical, microbiological, and sensory characteristics of milk, cream, and Colonial cheese was done, while cheese microbiota were characterized during ripening through metagenomic sequencing of the V4 region of the 16S rRNA gene. EOB supplementation reduced lipid oxidation during cheese maturation, with lower thiobarbituric acid reactive substances (TBARS) values observed after 45 days of ripening. This improvement in oxidative stability was achieved without major negative effects on the sensory characteristics of the products. The treatment did, however, modify the fatty acid composition, with an increase in saturated fatty acids and a decrease in the proportion of monounsaturated fatty acids. Despite these changes, sensory evaluations did not identify significant differences in flavor, texture, or overall acceptability of the milk, cream, or cheese, suggesting that the aromatic components associated with the essential oil treatment remained below the levels required to produce a detectable sensory effect. Metagenomic analysis provided additional information on the potential effects of the treatment on the cheese microbiota. EOB supplementation was associated with changes in the relative abundance of bacterial groups during ripening, including a reduction in some microorganisms considered potentially undesirable, while the overall richness and diversity of the bacterial community were maintained. The outcomes suggest that dietary supplementation with essential oil blends may influence both the oxidative stability and microbial ecology of cheese, although further work is needed to establish the mechanisms underlying these effects and their relevance under different production conditions.

In Contribution 6, Hu et al. combined rheological and tribological measurements to investigate the texture and oral processing characteristics of ambient sterilized yogurt. Instrumental approaches that can complement sensory evaluation are becoming increasingly relevant for the characterization of complex food textures. Ambient yogurt can develop undesirable textural characteristics, including graininess or grittiness, as a consequence of the high-temperature treatments required to obtain commercial shelf stability. Nine commercial yogurts with different microstructures and particle size distributions were compared, where rheological measurements were combined with tribological tests using a plate-contact tribometer and an artificial saliva containing mucin and α-amylase. The results showed that bulk rheological properties, including flow behavior and apparent viscosity, were mainly associated with sensations such as thickness and slipperiness. In contrast, the perception of graininess was more closely related to friction and lubrication occurring at the thin interfacial layer between the food and the contacting surfaces. This distinction highlights the complementary information provided by bulk rheology and oral tribology in the study of food texture [6]. The plate-contact configuration also provided greater sensitivity to the presence of particle aggregates associated with the perception of grittiness than conventional ball-on-plate measurements. These findings support the use of tribological measurements as an instrumental approach for investigating sensory attributes that are difficult to characterize through conventional rheological analysis alone [7]. More generally, linking instrumental measurements with sensory responses may help food manufacturers better predict and control mouthfeel during product development.

## 3. Conclusions and Future Research Perspective

In conclusion, the six contributions cover different points of the dairy production chain, from animal nutrition and milk quality to processing, microbiological control, and sensory characterization. Together, they illustrate how interventions at different stages can influence the quality, safety, and functionality of the final dairy products. In general, they emphasize the importance of connecting processes occurring at the farm, during processing, and at the point of consumption. The animal nutrition papers indicate that dietary supplementation with essential oils can modify milk composition and influence the oxidative stability of dairy products while maintaining acceptable sensory properties. Concerning the processing level, the use of inulin and probiotic or prebiotic ingredients provides opportunities to develop dairy products with modified nutritional profiles and additional functional properties. At the analytical level, multiplex qPCR and tribological approaches offer complementary tools for more rapid microbiological assessment and objective characterization of food texture. Despite these advances, several aspects deserve further investigation: (i) the essential oil long-term effect on animal rumen microbiota, animal performance, overall health; (ii) integration of multiplex qPCR with omics approaches (i.e., peptidomics, volatilomics) improving the understanding of microbial spoilage processes and enhance the prediction of dairy product shelf life; (iii) in vivo studies and clinical trials to confirm whether the functional effects observed in vitro translate into meaningful benefits for consumers; (iv) standardizing oral tribology models by accounting for salivary, enzymatic, thermal, and individual oral-processing variability to potentially improve the accuracy of in vitro predictions of food mouthfeel.

## Data Availability

No new data were created or analyzed in this study. Data sharing is not applicable to this article.
